# Ultrathin, Transparent, and High Density Perovskite Scintillator Film for High Resolution X‐Ray Microscopic Imaging

**DOI:** 10.1002/advs.202200831

**Published:** 2022-04-28

**Authors:** Xiaochen Wu, Zhao Guo, Shuang Zhu, Bingbing Zhang, Sumin Guo, Xinghua Dong, Linqiang Mei, Ruixue Liu, Chunjian Su, Zhanjun Gu

**Affiliations:** ^1^ CAS Key Laboratory for Biomedical Effects of Nanomaterials and Nanosafety and CAS Center for Excellence in Nanoscience Institute of High Energy Physics and National Center for Nanoscience and Technology Chinese Academy of Sciences Beijing 100049 China; ^2^ College of Mechanical and Electronic Engineering Shandong University of Science and Technology Qingdao 266590 China; ^3^ Fujian Key Laboratory of Translational Research in Cancer and Neurodegenerative Diseases Institute for Translational Medicine The School of Basic Medical Sciences Fujian Medical University Fuzhou 350122 China; ^4^ Center of Materials Science and Optoelectronics Engineering College of Materials Science and Optoelectronic Technology University of Chinese Academy of Sciences Beijing 100049 China; ^5^ Beijing Synchrotron Radiation Facility Institute of High Energy Physics Chinese Academy of Sciences Beijing 100049 China

**Keywords:** perovskite nanocrystals, resolution, scintillator film, X‐ray microscopic imaging

## Abstract

Inorganic perovskite quantum dots CsPbX_3_ (X = Cl, Br, and I) has recently received extensive attention as a new promising class of X‐ray scintillators. However, relatively low light yield (LY) of CsPbX_3_ and strong optical scattering of the thick opaque scintillator film restrict their practical applications for high‐resolution X‐ray microscopic imaging. Here, the Ce^3+^ ion doped CsPbBr_3_ nanocrystals (NCs) with enhanced LY and stability are obtained and then the ultrathin (30 µm) and transparent scintillator films with high density are prepared by a suction filtration method. The small amount Ce^3+^ dopant greatly enhances the LY of CsPbBr_3_ NCs (about 33 000 photons per MeV), which is much higher than that of bare CsPbBr_3_ NCs. Moreover, the scintillator films made by these NCs with high density realize a high spatial resolution of 862 nm thanks to its thin and transparent feature, which is so far a record resolution for perovskite scintillator‐based X‐ray microscopic imaging. This strategy not only provides a simple way to increase the resolution down to nanoscale but also extends the application of as‐prepared CsPbBr_3_ scintillator for high resolution X‐ray microscopic imaging.

## Graphical abstract

Ultrathin and transparent scintillator films with high density are prepared by a suction filtration method using Ce^3+^‐doped CsPbBr_3_ nanocrystal with high light yield and stability. Moreover, the scintillator films realize a record spatial resolution for perovskite scintillator‐based X‐ray microscopic imaging. This work extends the applications of as‐prepared CsPbBr_3_ scintillator for high‐resolution X‐ray microscopic imaging.

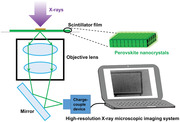

## 1 Introduction

X‐ray microscopic imaging technology is widely used in a variety of fields to accurately detect the internal tiny structure of objects in a nondestructive way, such as materials research, life sciences, natural resources, and microelectronics.^[^ ^1^, ^2^, ^3^, ^4^, ^5^, ^6^ ^]^ In typical X‐ray microscopic imaging systems (micro‐computed tomography (CT) and nano‐CT), scintillator sheet (5–50 µm thick) is located between the sample and objectives lens to achieve high spatial resolution down to 1 µm.^[^ ^7^, ^8^ ^]^ An ideal scintillator for X‐ray microscopic imaging system should have characteristics of thin thickness, high radioluminescence (RL) intensity and transparency, where thin scintillator can better match the depth of focus of the objective lens for high image quality,^[^ ^2^, ^9^, ^10^ ^]^ high RL intensity ensures that even thin scintillator film could also show bright light output and the good transparency can reduce the scattering of light and thus enhance the quality of image.^[^ ^11^, ^12^ ^]^ Currently, the commercial scintillators for X‐ray microscopic imaging systems are traditional single crystal scintillators, for example cerium‐doped lutetium‐aluminum garnet (LuAG:Ce),^[^ ^13^ ^]^ thallium‐doped cesium iodide (CsI:TI)^[^ ^14^, ^15^ ^]^ and cerium‐doped lutetium yttrium orthosilicate (LYSO:Ce) and so on.^[^ ^16^, ^17^, ^18^ ^]^ However, the current top‐down approach of preparing thin scintillators by grinding these bulk single crystal scintillators into sheets make the manufacture time‐ and cost‐ineffective. So far, it is of great difficulty to obtained the ultrathin single crystal scintillators below 50 µm. Alternatively, the bottom to up approach may provide a better opportunity for the fabrication of ultrathin and high‐quality scintillators, not only for easy preparation but also for better performance.

Perovskite nanocrystals (NCs), including CsPbBr_3_ NCs,^[^ ^19^, ^20^, ^21^, ^22^, ^23^, ^24^ ^]^ MAPbBr_3_ NCs (MA = CH_3_NH_3_^+^),^[^ ^25^, ^26^ ^]^ and so on,^[^ ^27^, ^28^, ^29^, ^30^, ^31^, ^32^, ^33^, ^34^, ^35^ ^]^ have displayed great potential as a new promising class of scintillators because of their many advantages such as strong X‐ray absorption ability, high RL intensity, fast light decay, and low‐cost solution synthesis. More importantly, compared with the big single crystal scintillators,^[^ ^36^, ^37^, ^38^, ^39^, ^40^, ^41^, ^42^, ^43^ ^]^ perovskite NCs scintillators are suitable for preparing thin scintillator films by using the promising bottom to up method due to their small sizes, quantum size effect, and tunable electronic bandgaps in the visible range. For example, many perovskite NCs‐based scintillators screen have been developed and show the potential for X‐ray imaging with the advantage of low‐cost, easy for preparation, and high detection sensitivity.^[^ ^12^, ^19^, ^22^, ^24^, ^30^, ^44^ ^]^ However, many scintillator films preparation methods^[^ ^23^, ^31^, ^45^, ^46^, ^47^, ^48^ ^]^ require the addition of auxiliary film‐forming geopolymers, which causes the low equivalent density of scintillator material in the film and thus results in the low RL intensity. To ensure the enough light output, the thick film is required but is usually opaque, which greatly increase the light scattering and seriously reduce the imaging resolution. As a result, the current X‐ray imaging using perovskite NCs scintillator films possess no advantage when compared with the micrometer resolution achieved in commercial micro‐CT imaging devices, where single‐crystal was used as the scintillator. There is still a challenge to balance the thickness, increase transparence and RL intensity of perovskite NCs scintillator films for its application in high resolution X‐ray microscopic imaging.

To address above issues, we developed a novel Ce^3+^ ion‐doped CsPbBr_3_ NCs to enhance its light yield (LY) and radiation stability, and the ultrathin (30 µm) and transparent scintillator films with high density based on these NCs were prepared by a suction filtration method. On the one hand, Ce^3+^ ion doping (8%) greatly enhances the LY of CsPbBr_3_ NCs, which is more than two times than the bare CsPbBr_3_ NCs. On the other hand, thanks to its high density of CsPbBr_3_ NCs in the film, the RL intensity of the scintillator film prepared by this method is about 10 times stronger than that prepared by spin‐coated method with the same thickness. As a result, our CsPbBr_3_ NCs scintillator film exhibiting strong RL intensity, ultrathin thickness, and high transparency enable X‐ray images with very high spatial resolution (≈862 nm), which is the highest resolution of reported perovskite NCs scintillator used for X‐ray imaging so far. Basing on this X‐ray microscopic imaging system, the nucleus of the onion epidermal cells and the circuits inside the chip were successfully observed. This work may open the way for perovskite NCs to replace the traditional single crystal scintillators in the field of high‐resolution X‐ray microscopic imaging.

## 2 Results and Discussion

**Figure 1:**
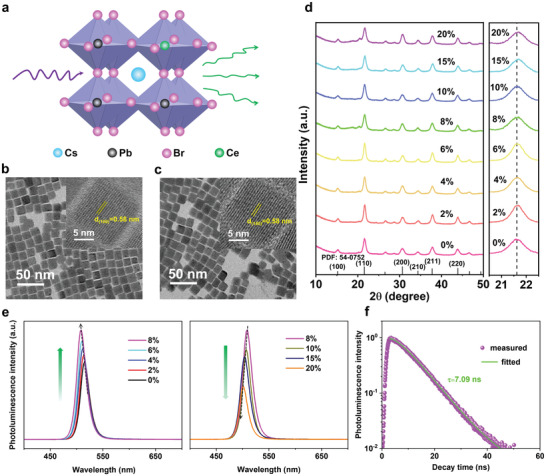
Crystal structure and photoluminescence (PL) characterization of Ce^3+^‐doped CsPbBr_3_ NCs. a) Crystal structure of the Ce^3+^ doped CsPbBr_3_ NCs. b) TEM image of Ce^3+^‐doped (8%) CsPbBr_3_ NCs and corresponding HRTEM image (top right). c) TEM image of CsPbBr_3_ NCs and corresponding HRTEM image (top right). d) Powder XRD pattern of the undoped and Ce^3+^‐doped CsPbBr_3_ NCs. e) PL spectra of the CsPbBr_3_ NCs with different Ce^3+^ ions content. f) PL decay curve of Ce^3+^‐doped (8%) CsPbBr_3_ NCs.

Traditional structural formula of perovskite is ABX_3_‐type, where “B” is the cation that has six anions of “X” and A is the cation that locates in a cavity formed by eight corner‐sharing BX octahedral.^[^ ^33^ ^]^ The replacement of B is generally considered as an effective method to enhance the photoluminescence (PL) intensity of perovskite scintillator NCs.^[^ ^49^, ^50^, ^51^, ^52^, ^53^, ^54^, ^55^, ^56^ ^]^ Here, we employed Ce^3+^ to enhance the PL intensity of CsPbBr_3_ NCs through a hot‐injected method.^[^ ^20^ ^]^ As shown in **Figure** **1**a, Pb^2+^ ions (B from ABX_3_) were replaced with Ce^3+^ ions during the synthetic process. As revealed by transmission electron microscopy (TEM), the as‐synthesized Ce^3+^‐doped CsPbBr_3_ NCs exhibit cubic shape (Figure 1b), which is similar to the pure CsPbBr_3_ NCs (Figure 1c). The high‐resolution TEM (HRTEM) images (top right insets in Figure 1b,c) revealed that both the doped and bare CsPbBr_3_ NCs were highly crystalline, and the lattice fringe is about 0.58 nm which is corresponded to the (100) plane of cubic CsPbBr_3_. The distribution of particle size (Figure S1, Supporting Information) and its statistical results (Figure S2, Supporting Information) demonstrated that with adding Ce^3+^ from 0% to 20%, the as‐prepared NCs had slight changes (±1 nm) in size. This proved that doped Ce^3+^ ions did not influence the crystal structure of CsPbBr_3_. The X‐ray powder diffraction (XRD) patterns of as‐prepared samples as shown in Figure 1d also reveals all peaks matched well with the cubic structure of CsPbBr_3_ (JCPDS No. 54‐0752) and Ce^3+^‐doping did not change the crystal structure of CsPbBr_3_ NCs hosts. Moreover, with increasing the Ce^3+^ content, the XRD peaks shift to a higher diffraction angle, further conforming the successful doping of Ce^3+^ into the CsPbBr_3_ NCs host matrix because the smaller ionic radius of dopant (Ce^3+^, 103.4 pm; Pb^2+^, 119 pm) usually induce the shift to high angel direction. We also characterized CsPbBr_3_ NCs with 8% Ce^3+^ by the TEM mapping (Figure S3, Supporting Information) and the energy‐dispersive X‐ray spectroscopy analysis (EDS) (Figure S4, Supporting Information). Both the TEM element mapping images and EDS results showed the existence of Ce^3+^ in the CsPbBr_3_ NCs. Next, we study the influence of different Ce^3+^‐doping concentrations on the optical properties of the CsPbBr_3_ NCs by PL spectra. As shown in Figure 1e, the PL intensity rapidly intensifies when the Ce^3+^ concentration increase from 0% to 8% and then weakens with further doping amount of Ce^3+^. Meanwhile, due to a slight adjust of the bandgap of NCs by Ce^3+^‐doping, the PL peaks of doped CsPbBr_3_ NCs also show a blueshift with increasing the content of Ce^3+^ ions. The PL decay time is another important feature for scintillators. As shown in Figure 1f, the decay curve of CsPbBr_3_ NCs doped with 8% Ce^3+^ shows a fast scintillation decay time (*τ* = 7.09 ns), which is similar to the previously reported CsPbBr_3_ NCs (*τ* = 7 ns).^[^ ^20^ ^]^ Both the high PL efficiency and fast scintillation decay time imply that the as‐prepared perovskite materials have the potential to function as effective scintillators.

**Figure 2:**
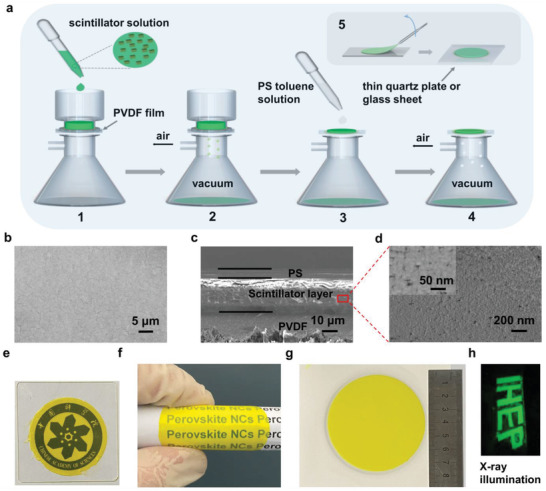
Preparation and characterization of scintillator film. a) Scheme of the scintillator film synthesis procedure by the suction filtration method. b) Top view of the scintillator film without PS protective layer. c) SEM image of the side scintillator film with PVDF substrate and d) its enlarged view. e) Photograph of the scintillator film on the top of the logo. f) Photographs of the flexible scintillator film and g) a large area scintillator film (50 cm^2^). h) Photograph of the “IHEP” logo under X‐ray illumination.

Due to its ultrasmall size (≈15 nm), the perovskite NCs scintillator films for X‐ray imaging are usually prepared by perovskite NCs solution with the advantage of low cost and easy manufacture. But the current preparations cannot realize the synthesis of ultrathin thickness, strong RL and high transparency of scintillator film for high‐resolution X‐ray microscopic imaging as mentioned above. In order to endow the application of perovskite NCs scintillator for high‐resolution X‐ray microscopic imaging, we developed a new “suction filtration” strategy to prepare scintillator films as shown in **Figure** **2**a. First, the solution of perovskite NCs was filtered by a polyvinylidene fluoride (PVDF) film (Figure S5, Supporting Information) using a vacuum pump to form a uniform scintillator layer with high density and ultrathin thickness on the PVDF film surface. Then, the scintillator layer was covered by a polystyrene (PS) layer to fill the gap between scintillator particles. Finally, the scintillator film could be transferred to ultrathin quartz sheet easily by tweezer. The thickness can be easily adjusted by changing the concentration and amount of perovskite NCs solution.

Next, to assess its potential as X‐ray scintillator film, we conducted various experiments to measure its structure, thickness, and transparency. For better evaluation of the performance of as‐prepared NCs film, we compared it with the scintillator film prepared by the normal spin‐coated method (Figure S6a, Supporting Information) with same thickness. The surface image of perovskite scintillator films prepared by suction filtration method shows that the perovskite NCs has a smooth surface and uniform scintillator film (Figure 2b). Moreover, through the side image of scintillator layer (of about 30 µm thickness, Figure 2c) and its enlarged view (Figure 2d), we can see the stacking of aggregates of scintillator nanocubes, proving the scintillator layer is dense. By contrast, we characterized the surface and side of the scintillator film prepared by the spin‐coated method. As the SEM images (Figure S6b, Supporting Information) shows, there are many tiny pores on the surface of the film, which may be caused by the rapid evaporation of solvent during the film forming process. In addition, from the side image of the film (Figure S6c, Supporting Information) and its enlarged view (Figure S6d, Supporting Information), the film consists of a large number of PS with only a small amount of scintillator nanoparticles, and there are many tiny cracks on the side of the film. Furthermore, we also used element mapping to show the different NCs density obtained by different method. As shown in Figure S7 (Supporting Information), density signals from Pb, Cs, and Br were observed in the core section of CsPbBr_3_ NCs film prepared by suction filtration method (Figure S7a, Supporting Information), while less density of Pb, Cs, and Br were found in the element mapping images of the film prepared by spin‐coated method (Figure S7b, Supporting Information). Therefore, the equivalent density of the scintillator film prepared by the spin coating is much lower than that of the film prepared by suction filtration method, which may lead to lower RL intensity under same X‐ray irradiation. Finally, we compared the transparency of scintillator films with the same thickness prepared by the two methods. We placed the logo of Chinese Academy of Sciences under the perovskite scintillator film on an ultrathin quartz sheet. As shown in Figure 2e, the details (such as Chinese and English characters) of the logo can be clearly seen through the scintillator film synthesized by suction filtration method, but only a fuzzy outline of the logo can be seen through the scintillator film prepared by the spin‐coated method (Figure S6e, Supporting Information). In addition, we also test its flexibility by bending the prepared scintillator film at a larger angle (Figure 2f; and Figure S8, Supporting Information), which proved that the scintillator film has good flexibility as well as stable luminescence performance at different bending angles. This may enable its application in flexible image sensor in the future. Furthermore, the large area is another important property in scintillator film for the preparation of photoelectric devices and X‐ray imaging systems. Using the suction filtration method, as is shown in the Figure 2g, a large area (50 cm^2^) scintillator film was prepared. It is worth mentioning that prepared scintillator films can be also cut into various shapes to meet different needs. As shown in Figure 2h, scintillator films are cut into “IHEP” logo, which yield strong and uniform green fluorescence under X‐ray excitation. Therefore, we prepared a uniform, large‐area, ultrathin, high‐density, and transparent perovskite scintillator film using this suction filtration method, which may greatly benefit its application for high‐resolution X‐ray microscopic imaging.

**Figure 3:**
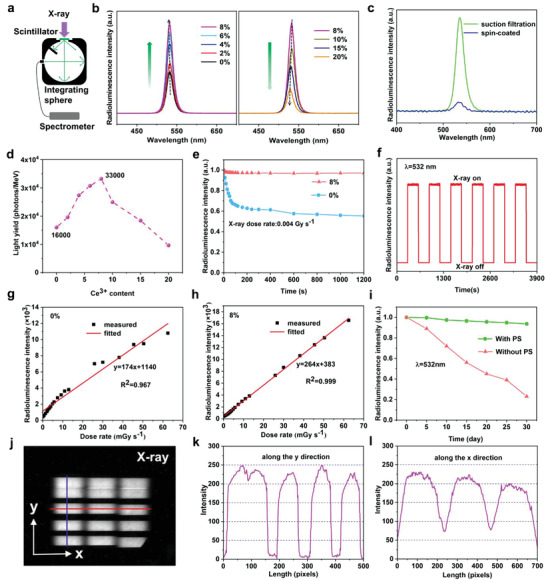
Radioluminescence (RL) characterization of as‐prepared scintillator film. a) Schematic of the testing system for RL intensity measurement. b) RL spectra of CsPbBr_3_ NCs with different Ce^3+^ doping concentrations. c) RL spectra of the scintillator films prepared by spin‐coated and suction filtration methods in the same thickness. d) Light yield of CsPbBr_3_ NCs with different Ce^3+^ doping concentrations. e) Radiation stability of CsPbBr_3_ NCs and CsPbBr_3_ with 8% Ce^3+^ doping concentration under continuous X‐ray irradiation for 1200 s and f) repeated cycles of X‐ray excitation with a time interval of 300 s. RL intensity measurements for g) undoped and h) Ce^3+^‐doped (8%) CsPbBr_3_‐based scintillator films as a function of different X‐ray dose rates. i) Environmental stability of scintillator film with PS protection layer within a month. j) RL responses of the scintillator film to Beijing Sychrotron Radiation Facility (BSRF). The change of gray value along the k) y direction and l) x direction of j).

Encouraged by its ultrathin thickness, high density, and transparency, we next evaluate the RL performance of as‐prepared scintillator film using an integrating sphere test (**Figure** **3**a). The RL intensity, stability, and uniformity are systematically studied. Firstly, similar with the results from PL measurement of the solution test, the scintillator film with 8% Ce^3+^ doped exhibit the best RL intensity (Figure 3b). As shown in Figure 3c, the RL intensity of scintillator film prepared by suction filtration method is about 10 times higher than that prepared by spin‐coated method with the same thickness because of its high density and transparency. Moreover, LY that describes the internal X‐ray‐to‐photon conversion efficiency determines the RL intensity. We then measured the LY of CsPbBr_3_ NCs with various Ce^3+^ contents (Figure 3d), where the CsPbBr_3_ NCs with 8% Ce^3+^ doping content delivered a highest LY of 33 000 photons per MeV, much higher than the commercial LuAG:Ce (22 000 photons MeV).^[^ ^57^ ^]^ In our case, the LY is dependent on the doping amount of Ce^3+^ ions. The LY rapidly intensifies when the Ce^3+^ concentration increase from 0% to 8% and then weakens with further doping amount of Ce^3+^. It is shown that with the increasing doping amount of Ce^3+^ ions, the bandgaps of the CsPbBr_3_ NCs host gradually became larger which is consistent with the blueshift of PL and RL. As a factor responsible for the shift of PL/RL spectra, the quantum confinement effect induced by the size variation was eliminated by the size‐distribution analysis (Figure S2, Supporting Information). Whereas the doping of Ce^3+^ ions almost have no influence on its size distribution. Therefore, this effect can be regarded as a result of the doping‐induced state modification.^[^ ^56^, ^58^ ^]^ As defects, the introduction of Ce^3+^ ions may influence the optical bandgap of Cs‐Pb‐Br. It is believed that doping metal ions into the lattice will normally bring on a new near band‐edge states,^[^ ^59^, ^60^ ^]^ which increase the density of the lowest excitonic state, as highlighted by an asterisk in Figure S9 (Supporting Information). Filling of the CB with extra electrons donated by dopants will normally result in more band‐edge PL emissions (i.e., PL enhancement), and the influence of nonradiative trap states on this PL channel is low;^[^ ^56^ ^]^ In contrast, when the doping amount of Ce^3+^ increase further, the defect density also increases greatly, which may enable the trap states to play a dominant role and thus PL quenching would take place instead.^[^ ^59^ ^]^ This decrease in emission intensity suggests that much more Ce^3+^ ions may have been incorporated into the CsPbBr_3_ host lattice, inducing strong perturbation to the density of states. Such assertation was confirmed by the XRD results (Figure 1d). With the increasing dopant amount of Ce^3+^ ions, the peaks shift to higher angle, indicating the host lattices have been changed a little by dopants. Therefore, based on our results and previous reports, the dopants induces the defect state modification contribute to the different trend of LY with the change of Ce^3+^ doping amount.

In addition to the RL intensity, the X‐ray radiation, and environmental stability of scintillator is another important factor for its application in X‐ray imaging. We thus compared the photostability of undoped and 8% Ce^3+^ doping concentration of CsPbBr_3_ NCs under continuous X‐ray irradiation, respectively (Figure 3e; and Figure S11, Supporting Information). As the result goes, the RL intensity of 8% Ce^3+^ doped CsPbBr_3_ NCs showed no obvious reduction while undoped CsPbBr_3_ NCs attenuates to initial 55% after continuous X‐ray irradiation for 1200 s. Moreover, under repeated cycles of X‐ray, the scintillator film also showed good photostability (Figure 3f). It is well known that, in the most Pb halide perovskite, Pb^2+^ is prone to be reduced to metallic Pb^0^ upon heating or illumination.^[^ ^61^, ^62^, ^63^ ^]^ The formation of Pb^0^ severely reduces the performance of perovskite film and its long‐term durability.^[^ ^64^ ^]^ To solve this problem, many strategies have been developed and very recently, the use of Ce^3+^/Ce^4+[^ ^65^ ^]^ or Eu^3+^/Eu^2+[^ ^66^ ^]^ redox pairs have been found to eliminate the Pb^0^ formation by oxidizing Pb^0^ to Pb^2+^ and thus increase the stability of perovskite film. In our work, we try to use Ce^3+^ doping to inhibit the Pb° formation and thus increase its photostability. As shown in Figure S12 (Supporting Information), after 3 h X‐ray irradiation, the XRD pattern of undoped CsPbBr_3_ NCs film changed a little and a few new peaks appeared, which can be ascribed to Pb (PDF: 44‐0872). Moreover, the characteristic peaks of pure CsPbBr_3_ NCs become narrower after the test, which indicates the change of crystallinity of NCs. In contrast, the pattern of Ce^3+^‐doped (8%) CsPbBr_3_ NCs shows no change, confirming that Ce^3+^ doping could not only eliminate Pb^0^ defects formation but also increase its crystal stability. Following the photostability, the relationship of X‐ray dose and RL intensity of the scintillator film was further assessed. We recorded the RL spectra of both undoped scintillator film and 8% Ce^3+^ doped scintillator film under various X‐ray dose rates (Figure S13, Supporting Information) to evaluate its linear response property. As indicated by the results, pure CsPbBr_3_ NCs scintillator film tend to degrade under X‐ray (Figure S12, Supporting Information), thus leading to nonlinear RL response to X‐ray dose rate (Figure 3g). In contrast, due to Ce^3+^ ions’ ability to improve the radiative photostability of CsPbBr_3_ NCs, as shown in Figure 3h, the RL intensity of scintillator film with 8% Ce^3+^ doping concentration shows linear response even at high X‐ray dose rate. This unique characteristic makes the prepared scintillator film suitable for X‐ray detection application. Apart from its photostability, we next evaluate its environmental stability since CsPbBr_3_ NCs are usually unstable in the open condition. Thanks to the protection of PS layer which can cut off the oxygen and moisture, the scintillator films also showed a good environmental stability even after 1 month (Figure 3i).

Finally, the uniformity of the RL of scintillator films under X‐ray irradiation is also evaluated since it is an important factor for its application in X‐ray imaging. Therefore, we tested the RL uniformity of CsPbBr_3_:Ce^3+^ (8%) scintillator films on an ultrathin quartz substrate by the Beijing Synchrotron Radiation Facility (BSRF). As shown in Figure 3j, a synchrotron radiation source was used to test fifteen images at different locations of the scintillator film and stitched them together. It is verified that the RL intensity of the scintillator film varies little in different places. Then we measured the change of gray values in the X and Y directions of the X‐ray image. The results (Figure 3k,l) show that the gray value along the X direction and Y direction is almost the same, which indicates that the scintillator film is uniform and has a good response to synchrotron radiation sources. This feature also indicates its potential applications in high‐resolution synchrotron radiation X‐ray microscopic imaging.

**Figure 4:**
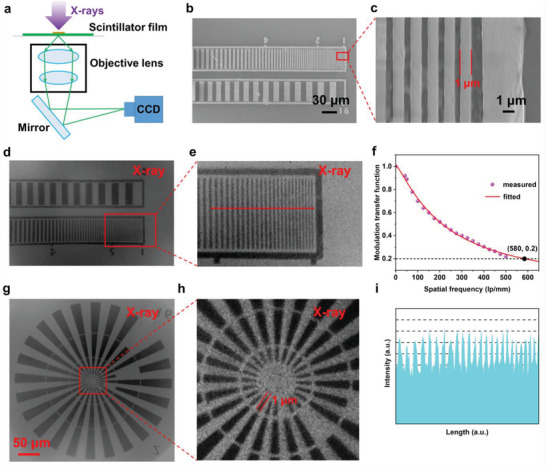
X‐ray microscopic imaging system and resolution. a) Schematic of the X‐ray microscopic imaging system. b,c) SEM images of the line pair card. d,e) X‐ray microscopic images of the line pair card and f) modulation transfer function (MTF) curve of the X‐ray microscopic imaging system using an objective lens with magnification of 40. The thickness of scintillator film used in this imaging system is 30 µm. g,h) The X‐ray microscopic images of the center area of the line pair card. i) The change of gray value along the line in e).

The above characterizations all indicate the potential of CsPbBr_3_ NCs film as scintillator for X‐ray imaging. Herein, we built a home‐made X‐ray microscopic imaging system that composed of X‐ray source, inverted microscope, and visible charge‐couple device (CCD) (Figure S14, Supporting Information). In order to obtain better imaging quality, the optimized scintillator film with 8% Ce^3+^ doping concentration and 30 µm of thickness were selected for this imaging system (Figure S15, Supporting Information). As shown in **Figure** **4**a, the perovskite scintillator film absorbs high‐energy X‐rays from the X‐ray tube and emits low‐energy visible light, which is detected by the CCD after passing through the objective lens of the inverted microscope. We then compared the image quality of the scintillator films with same thickness prepared by suction filtration and spin‐coated methods in this X‐ray microscopic imaging system, respectively. As expected, the image quality using the scintillator film prepared by spin‐coated method (Figure S16a, Supporting Information) is much lower than that obtained by the suction filtration method (Figure S16b, Supporting Information). Since the abrupt change in intensity can be reflected by the slope across the boundary, we thus used the edge spread functions to quantitatively measure and compare the image quality. As shown in Figure S16c,d (Supporting Information), the measured slope for the scintillator film prepared by suction filtration method was 129 gray value/pixel, which is much larger than the spin‐coated method (18.2 gray value/pixel). Furthermore, we used the line pair card (Figure 4b,c; and Figure S17, Supporting Information) to test the spatial resolution of this X‐ray microscopic imaging system. The X‐ray images of the line pair card show the high imaging quality and spatial resolution using an objective lens with a magnification of 40 (Figure 4d,e). Herein, the modulation transfer functions (MTF) that can quantify the spatial resolution was calculated. As presented in Figure 4f, the spatial resolution (defined as the spatial frequency value at MTF = 0.2) is 580 lp mm^−1^ (≈862 nm), which is the highest spatial resolution outcome among all the reported perovskite scintillator film to our best knowledge. The X‐ray images (Figure 4g,h) of the circular area of the line pair card (Figure S17c,d, Supporting Information) also show the high imaging resolution (below 1 µm) of under as‐prepared X‐ray microscopic imaging system. What's more, the sharp intensity change of gray values between the black and white line (along the red line in Figure 4e) also shows the high spatial resolution of the X‐ray microscopic imaging system (Figure 4i).

**Figure 5:**
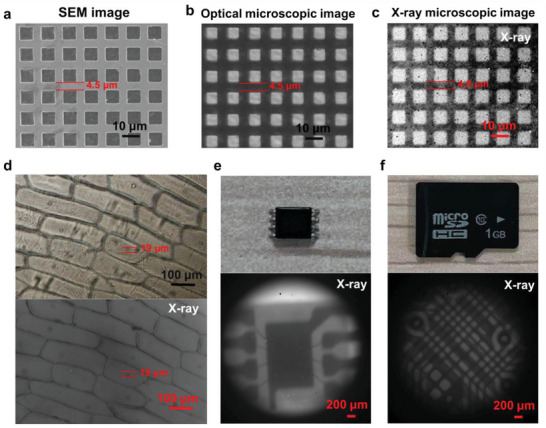
Application of this X‐ray microscopic imaging system. a) SEM image, b) optical microscopic image, and c) X‐ray microscopic image of the copper mesh. d) Onion epidermal cells stained with iodine solution of potassium iodide (top) and its X‐ray microscopic image (bottom). e) Photograph of the chip (top) and its X‐ray microscopic image (bottom). f) Photograph of the storage card (top) and its X‐ray microscopic image (bottom).

To assess the feasible applications of the X‐ray microscopic imaging system using the as‐prepared high‐performance perovskite scintillator film, experiments were carried out in several different areas. First, since ultrathin transparent perovskite scintillator films can be prepared by the suction filtration method, we believe that the X‐ray microscopic imaging system can achieve colocalization of optical microscopic imaging and X‐ray microscopic imaging. The SEM image of the copper mesh is shown in **Figure** **5**a, and its narrowest width is 4.5 µm. Due to the higher X‐ray absorption capacity of copper, the density difference cannot be determined from optical microscopic images (Figure 5b) alone, but X‐ray images (Figure 5c) can. By comparing the optical microscopic image and X‐ray microscopic image of the same position, we can get the difference of the density of the imaging object at different positions. Then, to demonstrate the biological application of this X‐ray microscopic imaging system, onion epidermal cells were stained using iodine solution of potassium iodide. As shown in the upper part of Figure 5d, the cytoplasm appears light yellow, while the nucleus is dark yellow. Due to the higher X‐ray absorption ability of nucleus, it could be distinctively identified by the X‐ray microscopic image from the dyed onion epidermal cells (lower part of Figure 5d). This result showed the potential application for cell structure identification with different density. Finally, in order to verify the applicability in the field of microelectronics, we performed X‐ray imaging on the chip and storage card (Figure 5e,f). The clear X‐ray images of the chip and storage card also show the potential in microelectronics. The above imaging demonstrations provide direct evidence that the perovskite scintillator films prepared by Ce^3+^‐doped CsPbBr_3_ NCs have great potential in various X‐ray microscopic imaging application.

## 3 Conclusions

In conclusion, we have successfully synthesized a series of Ce^3+^‐doped CsPbBr_3_ NCs that exhibit higher LY and stability under X‐ray irradiation. Then the ultrathin (30 µm) and transparent scintillator films with high density based on Ce^3+^‐doped (8%) CsPbBr_3_ NCs were prepared by a suction filtration method. Such ultrathin, transparent, and high RL intensity scintillator film further achieved the unprecedented imaging resolution to ≈862 nm in the field of perovskite NCs used for X‐ray imaging. The X‐ray imaging application results also show that the as‐prepared perovskite NCs have great potential in the field of X‐ray microscopic imaging, such as biological tissue imaging and microcircuit detection. Our study provides a novel method to obtain high‐performing scintillator films and further accelerate the application of perovskite NCs in high‐resolution X‐ray microscopic imaging.

## 4 Experimental Section

### Materials

Caesium carbonate (Cs_2_CO_3_, 99.9%), oleic acid (OA, technical grade 90%) were purchased from Sigma‐Aldrich. Lead(II) bromide (PbBr_2_, 99.998%), cerium(III) bromide (CeBr_3_, 99.9%), 1‐octadecene (ODE, technical grade 90%), methanol (CH_3_OH, 99.8%) were purchased from Alfa Aesar. Oleylamine (OAm, technical grade 90%) was purchased from Macklin. Toluene (C_7_H_8_, AR) was purchased from Beijing Chemical Works. Polystyrene (PS) was purchased from Acros. Polyvinylidene fluoride (PVDF) membrane with pore size of 0.2 µm was provided by Beyotime Biotechnology. All the chemicals were used as received without further purification.

### Characterization

TEM and high‐resolution TEM images were taken on a FEI Tecnai G2 F30 with an accelerating voltage of 300 kV. Elements mapping and the energy dispersive X‐ray spectroscopy (EDS) were acquired with an accelerating voltage of 300 kV. Powder X‐ray diffraction (XRD) characterization was measured using Bruker D8 Advance with Cu K*α* radiation (*λ* = 1.54 184 Å). SEM images were observed by scanning electron microscopy (SEM, Hitachi S‐4800). The photoluminescence (PL) spectra were obtained using a fluorescence spectrophotometer (Horiba Fluorolog‐3). PL lifetime experimental was performed on a Horiba Fluorolog‐3 with a Nano LED of 340 nm. The radioluminescence (RL) spectra were obtained using a fluorescence spectrophotometer (Horiba Fluorolog‐3), and a Mini‐X X‐ray tube (Amptek Inc.) was used as X‐ray source (50 kV, 75 µA). The relationship between RL intensity and dose rate was measured by the X‐ray source (MultiRad 160) and a portable optical fiber spectrometer (AvaSpec‐HS‐TEC).

### Synthesis of Cs‐Oleate As a Caesium Precursor

Cs_2_CO_3_ (0.4 g), OA (1.5 mL), and ODE (15 mL) were added into a 50 mL single‐neck round‐bottom flask. The mixture was heated to 100 °C under vacuum condition for 1 h. Then the mixture is heated to 150 °C under Argon condition until the Cs_2_CO_3_ powder totally dissolve. The Cs‐precursor solution was kept at 150 °C in an Argon condition before using in NCs synthesis.

### Synthesis of Ce‐Oleate Solution

CeBr_3_ (0.684 g) and methanol (10 mL) were loaded in to a 50 mL single‐neck round bottom flask. Then CeBr_3_ powder was dissolved by ultrasound for 10 min. OA (10 mL) was added into flask and the mixture was then heated to 80 °C under Argon condition to totally remove methanol. The clear Ce‐oleate solution was stored at room temperature.

### Synthesis of CsPbBr_3_ NCs

PbBr_2_ (0.66 g), OAm (5 mL), OA (5 mL), and ODE (50 mL) were added into a 250 mL three‐neck round‐bottom flask. The mixture was heated to 100 °C under Argon condition for 30 min. Then the mixture was heated to 160 °C to totally dissolve the PbBr_2_ powder. 5 mL hot Cs‐oleate precursor solution was injected quickly into the flask. After 10 s of reaction, the flask was put into an ice bath and the as‐prepared CsPbBr_3_ NCs was stored at room temperature.

### Synthesis of Ce^3+^‐Doped CsPbBr_3_ NCs

According to different doping concentrations (*x* = 2%, 4%, 6%, 8%, 10%, 15%, and 20%), Ce‐oleate and PbBr_2_ (total 1.8 mmol), OAm (5 mL), OA (5 mL), and ODE (50 mL) were added into a 250 mL three‐neck round‐bottom flask. The mixture was heated to 100 °C under Argon condition for 30 min. Then the mixture was heated to 160 °C to totally dissolve the PbBr_2_ powder. 5 mL hot Cs‐oleate precursor solution was injected quickly into the flask. After 10 s of reaction, the flask was put into an ice bath and the as‐prepared Ce^3+^‐doped CsPbBr_3_ NCs was stored at room temperature.

### Preparation of Scintillator Films Using Suction Filtration Method

All instruments were rinsed with ethanol and deionized water prior to preparation of scintillator film. Scintillator films of different sizes can be prepared from different sizes of suction bottles. A filter bottle containing a sand plate with a diameter of 1.5 cm was selected and PVDF membrane (2 × 2 cm^2^) was placed on the sand core. Add 1 mL scintillator solution (25 mg mL^−1^) into the filter bottle, start the vacuum pump and make the vacuum degree reach 0.01 MPa. After 10 min, turn off the vacuum pump, when scintillator particles evenly accumulate on PVDF membrane. Toluene solution of PS (50 mg mL^−1^) was add to scintillator layer until the scintillator layer was fully covered. Start the vacuum pump until all the toluene falls into the extraction bottle. Turn off the vacuum pump and remove the scintillator film with PVDF substrate from the suction flask. The thickness of prepared scintillator layer is about 30 µm.

### Preparation of Scintillator Films Using Spin‐Coated Method

1000 mg of PS was dissolved in 10 mL toluene to obtained a 100 mg mL^−1^ PS solution. Perovskite powder (500 mg) was added in 10 mL of the above solution and the mixture was uniformly dispersed by ultrasound for 10 min. Finally, the scintillator film was formed by dropping the mixture onto a glass slide, with spin‐coating speed at a 1200 rpm.

### Light Yield Measurements

Light yield was measured by Horiba FluoroLog‐3 with integrating sphere (Figure 3a). X‐ray tube was set to 50 kV and 75 µA in all measurement and its output spectra are shown in Figure S10a (Supporting Information). The light yield of as‐prepared Ce^3+^‐doped CsPbBr_3_ NCs was calculated by compared to known light yield data of Ce‐doped LuAG scintillator (22 000 photons MeV). According to previous reported light yield calculated method,^[^ ^57^ ^]^ the Ce^3+^‐doped CsPbBr_3_ film and LuAG:Ce were placed on top port of integrating sphere with aluminum foil covered. RL spectra were then measured and shown in Figure S10b,c,d, Supporting Information. The emission photons of scintillator were normalized to the same X‐ray attenuation according to the following equation

(1) $$I_{\mathrm{normalized}}=\frac{I_{\mathrm{measured}}}{A}$$

where *A* is the equivalent X‐ray attenuation coefficient of scintillators from 2 to 50 KeV,^[^ ^67^ ^]^ *I*_normalized_ and *I*_measured_ are normalized and measured emission photons counts. Next, the light yield of Ce^3+^‐doped CsPbBr_3_ film can be calculated from the following equation

(2) $$LY_{\mathrm{CsPbBr}3}=LY_{\mathrm{LuAG}:\mathrm{Ce}}\times \frac{I_{\mathrm{CsPbBr}3}}{I_{\mathrm{LuAG}:\mathrm{Ce}}}$$

where *LY*_CsPbBr3_ and *LY*_LuAG:Ce_ is the light yield of Ce^3+^‐doped CsPbBr_3_ film and LuAG:Ce, *I*_CsPbBr3_, and *I*_LuAG:Ce_ are the normalized photon counts of Ce^3+^‐doped CsPbBr_3_ film and LuAG:Ce.

### RL Test of Scintillator Film Irradiated on BSRF

The RL of scintillator film was tested on BSRF. High energy X‐rays pass through the windows (10 mm aluminum + 0.5 mm aluminum) and irradiate the scintillator film. A detector (1024 × 1024, pixel size: 20 µm) is used to capture RL photograph of scintillator film. The imaging system has a magnification of 2.5 and the exposure time is 50 µs.

### X‐Ray Microscopic Imaging System

The X‐ray microscopic imaging system is consisted of Mini‐X X‐ray source, Fluorescent inverted microscope (Olympus IX73) and CCD Camera. Several Lead plates were placed around the X‐ray tube to shield radiation. The X‐ray pictures were captured by a cooling CCD camera (2048 × 2048 pixels, pixel size: 7.4 µm).

### MTF Measurements

On the X‐ray image of the line pair card, the gray value distribution curve which is perpendicular to the line direction is obtained by selecting the line pair group with the widest line. The difference between the maximum and minimum gray values of the width line pairs represents the actual contrast (Δµ). The difference between the maximum and minimum gray values of other line pair is calculated in turn, and the difference represents the effective contrast (Δµ_0_). Therefore, the modulation of each line pair can be expressed as [(Δµ_0_)/(Δµ)]. Then use logarithmic curve‐fitting to get the calculated resolution (LP/mm) when modulation is 20%.

## Conflict of Interest

The authors declare no conflict of interest.

## Supporting information

Supporting Information

## Data Availability

Research data are not shared.
