# Supplementary material for: Ultrathin, Transparent, and High Density Perovskite Scintillator Film for High Resolution X‐Ray Microscopic Imaging
Source: Adv Sci (Weinh). 2022 Apr 28;9(17):2200831. doi: 10.1002/advs.202200831 (PMC9189653; doi:10.1002/advs.202200831)
Supplement: Supplementary file 1 — Supporting Information [file ADVS-9-2200831-s001.pdf]

Supporting Information

Ultrathin, Transparent and High Density Perovskite Scintillator Film for High Resolution X-ray Microscopic Imaging

Xiaochen Wu[1,2#], Zhao Guo[3#], Shuang Zhu[1,4], Bingbing Zhang[5], Sumin Guo[2], Xinghua Dong[1], Linqiang Mei[1,4], Ruixue Liu[1], Chunjian Su[2*], Zhanjun Gu[1,4*]

[1]CAS Key Laboratory for Biomedical Effects of Nanomaterials and Nanosafety and CAS Center for Excellence in Nanoscience, Institute of High Energy Physics and National Center for Nanoscience and Technology, Chinese Academy of Sciences, Beijing 100049, China
[2]College of Mechanical and Electronic Engineering, Shandong University of Science and Technology, Qingdao 266590, China
[3]Fujian Key Laboratory of Translational Research in Cancer and Neurodegenerative Diseases, Institute for Translational Medicine, The School of Basic Medical Sciences, Fujian Medical University, Fuzhou 350122, China
[4]Center of Materials Science and Optoelectronics Engineering, College of Materials Science and Optoelectronic Technology, University of Chinese Academy of Sciences
[5]Beijing Synchrotron Radiation Facility, Institute of High Energy Physics, Chinese Academy of Sciences, Beijing 100049, China.
# These authors contributed equally to this work.

* Corresponding author: Chunjian Su, E-mail: suchunjian@sdust.edu.cn; Zhanjun Gu, E-mail: zjgu@ihep.ac.cn.

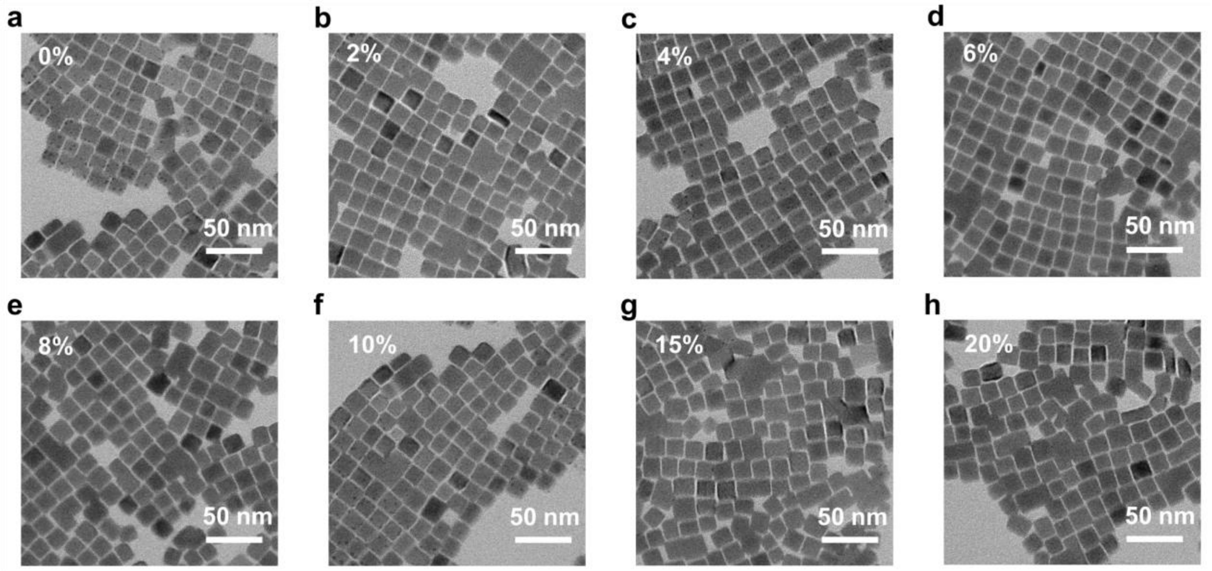

Figure S1. Morphology characterized by TEM. a-h) TEM images of $CsPbBr_3$ NCs with 0%, 2%, 4%, 6%, 8%, 10%, 15% and 20% Ce^{3+} doped.

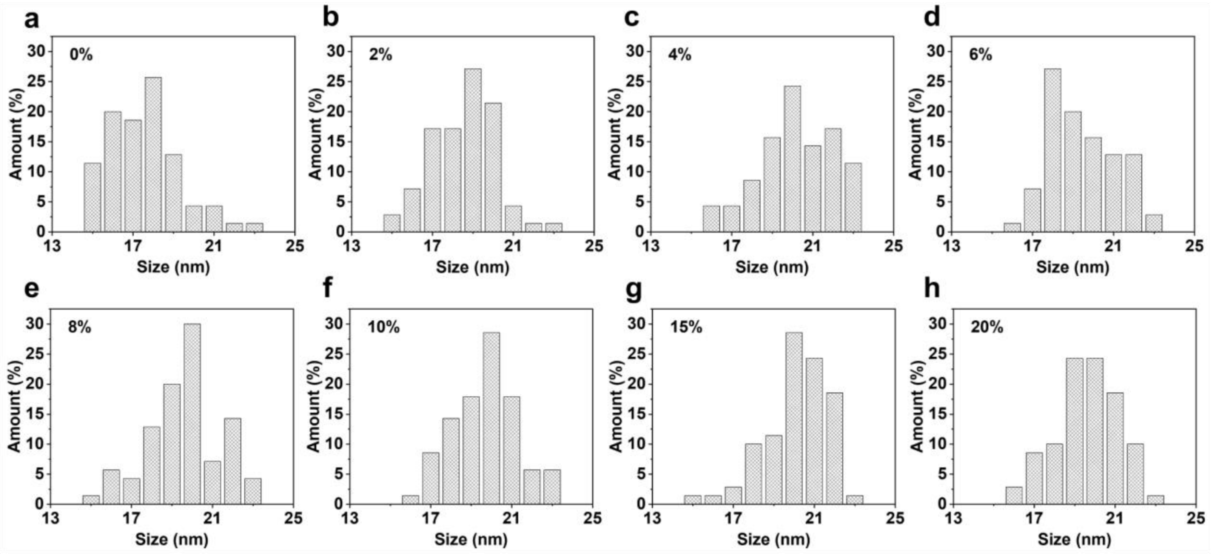

Figure S2. Statistical results of particle size. a-h) Statistical results of particle size distribution of $CsPbBr_3$ NCs with 0%, 2%, 4%, 6%, 8%, 10%, 15% and 20% Ce^{3+} doped.

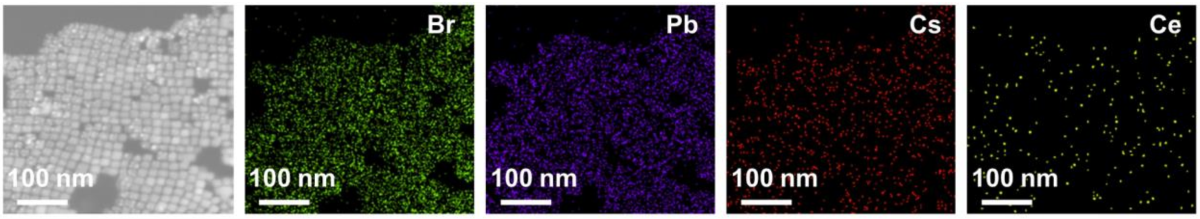

Figure S3. TEM mapping of $CsPbBr_3$ NCs with 8% Ce^{3+} doped.

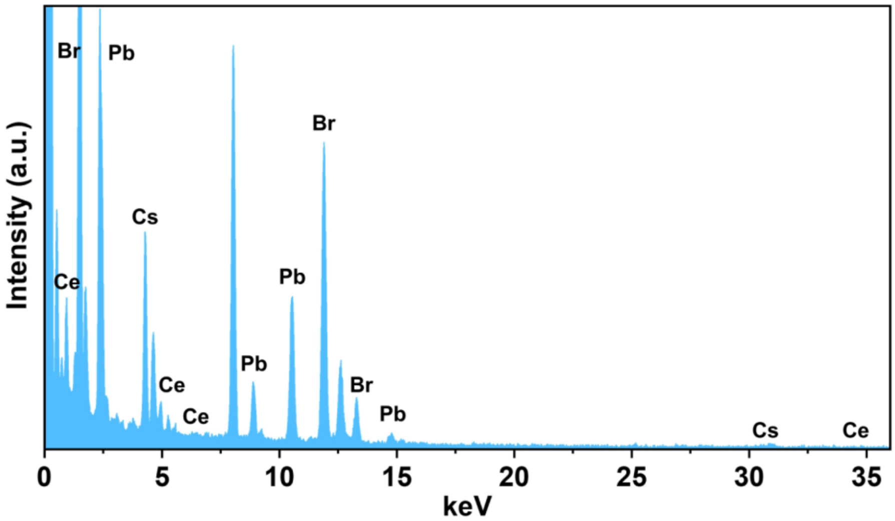

Figure S4. Energy-dispersive X-ray spectroscopy analysis (EDS) of $CsPbBr_3$ NCs with 8% Ce^{3+} doped.

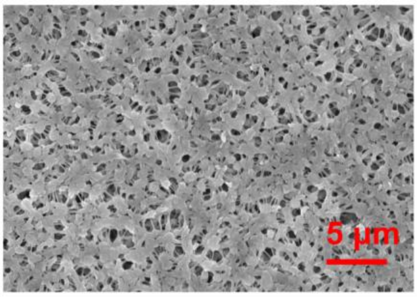

Figure S5. SEM image of PVDF membrane surface.

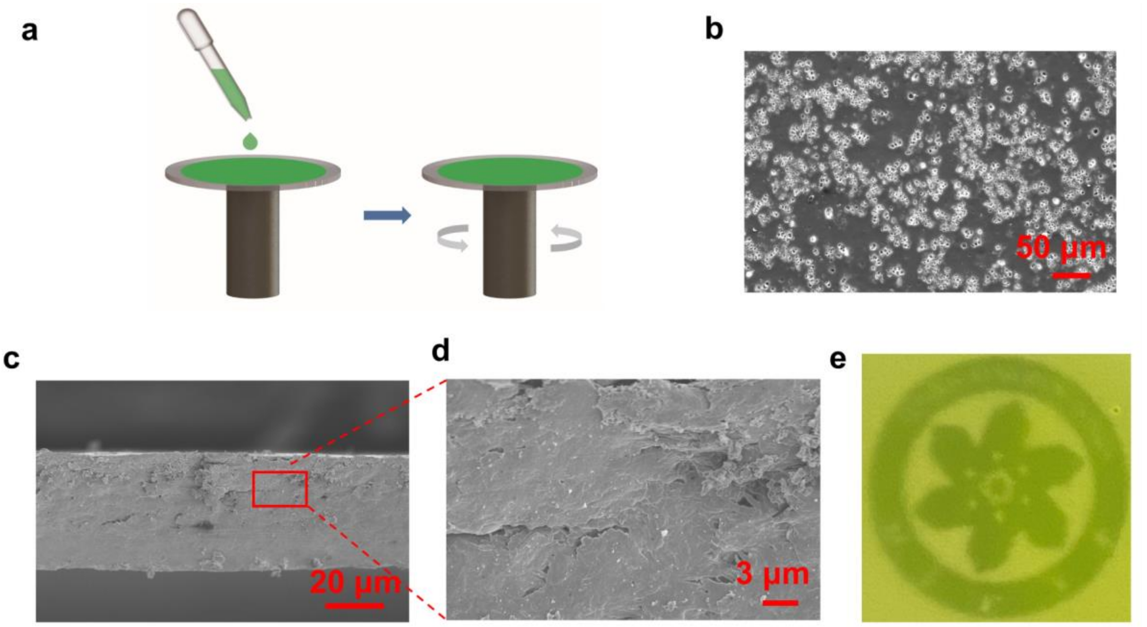

Figure S6. Scintillator film prepared by spin-coated method and its characterizations. a) Schematic diagram of scintillator film prepared by spin-coated method. The (b) surface , (c) side and (d) enlarged side SEM images of scintillator film prepared by spin-coated method. e) Photograph of the scintillator film on the top of the logo.

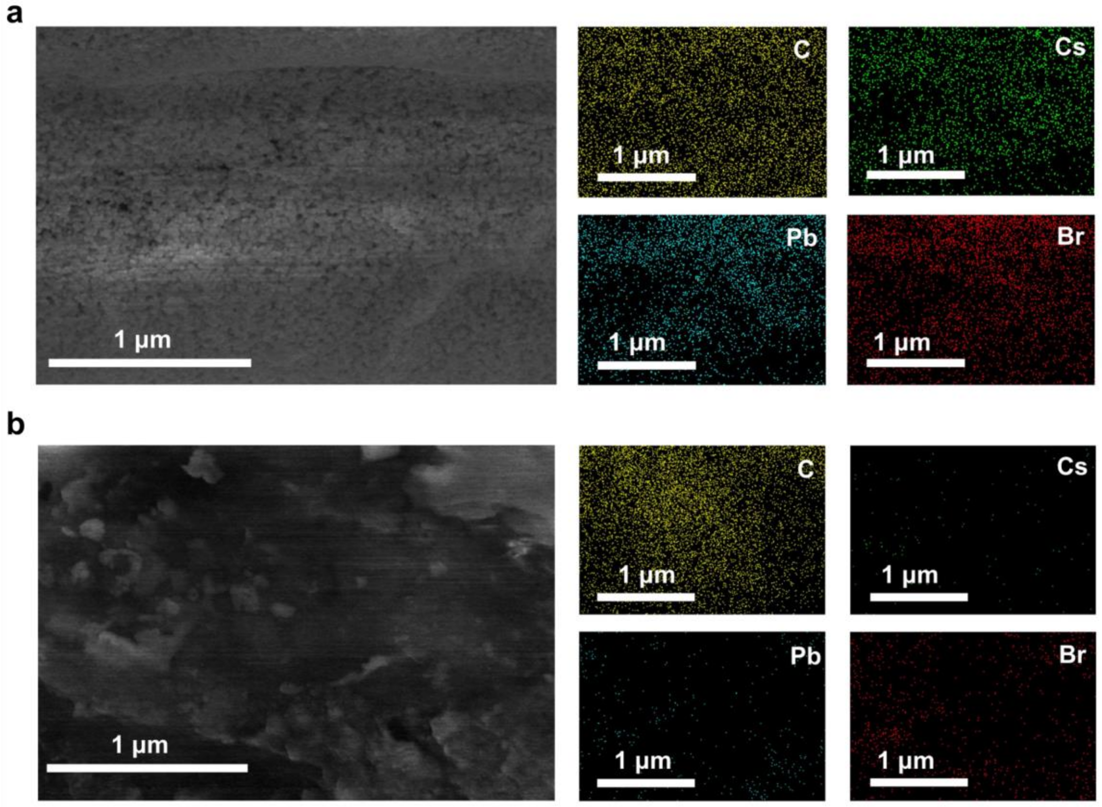

Figure S7. SEM element mapping images of the core section of scintillator films prepared by (a) suction filtration method and (b) spin-coated method.

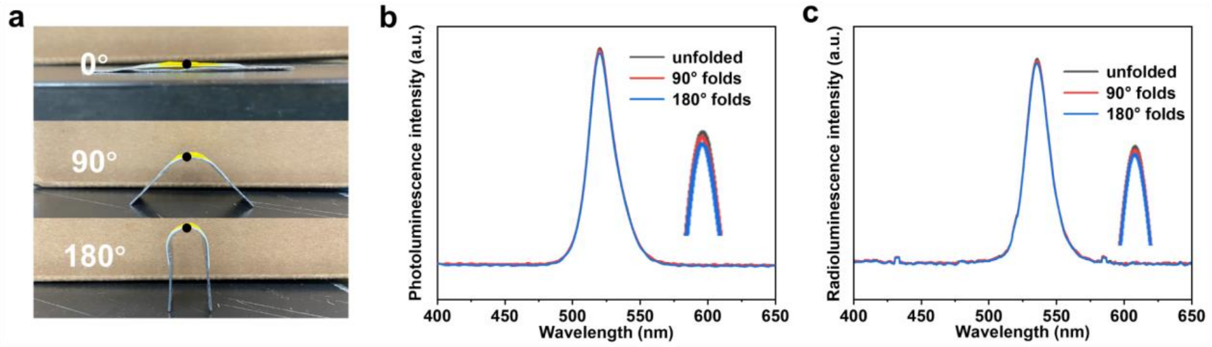

Figure S8. a) Photographs of scintillator films under 0°, right-angle outwards (90°) and 180° folds. b) Photolumninescence and (c) radioluminescence changes of a spot on scintillator film under 0°, right-angle outwards (90°) and 180° folds. The result showed that bending film does not affect its luminescence performance.

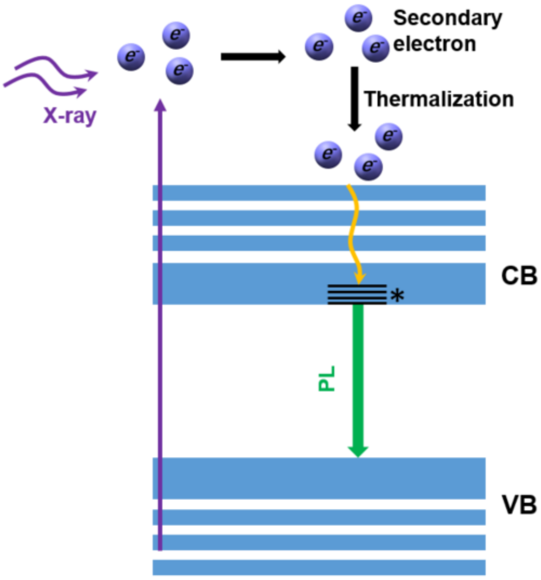

Figure S9. Schematic illustration of the RL mechanism of Ce^{3+} ion-doping under X-ray illumination, where VB and CB denote valence band and conduction band. The asterisk depicts the Ce^{3+}-doping-induced states near the CB band-edge.

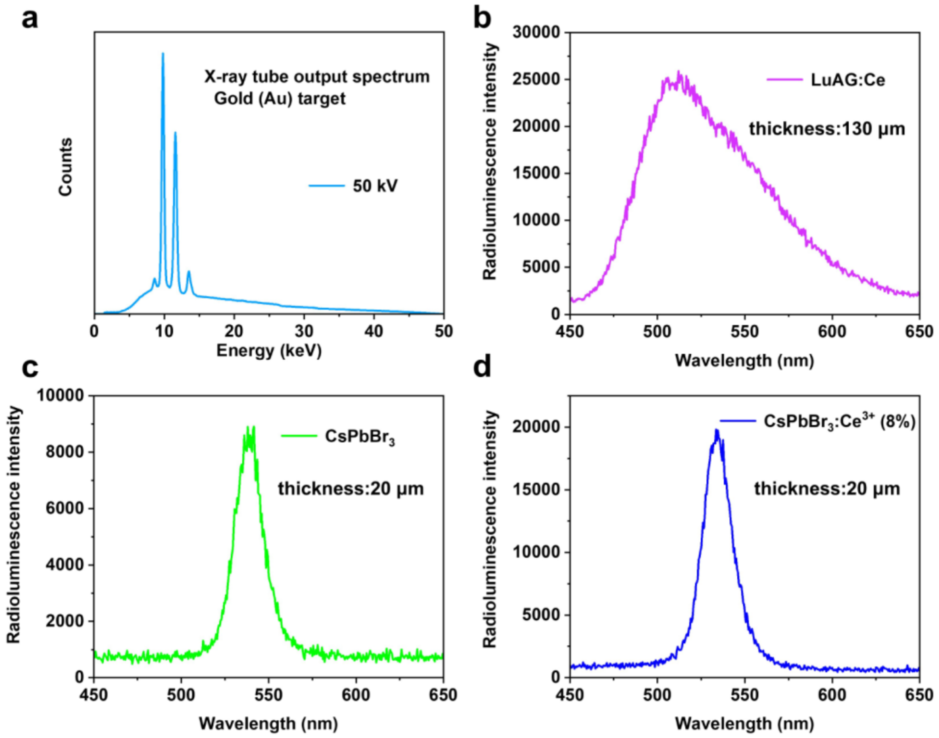

Figure S10. a) Output spectra of X-ray tube at 50 kV (target:Au). Radioluminescence spectra of (b) LuAG:Ce (thickness: 130 μm), c) $CsPbBr_3$ scintillator film (thickness: 20 μm) and (d) $CsPbBr_3:Ce^{3+}$ (8%) scintillator film (thickness: 20 μm) in this test system.

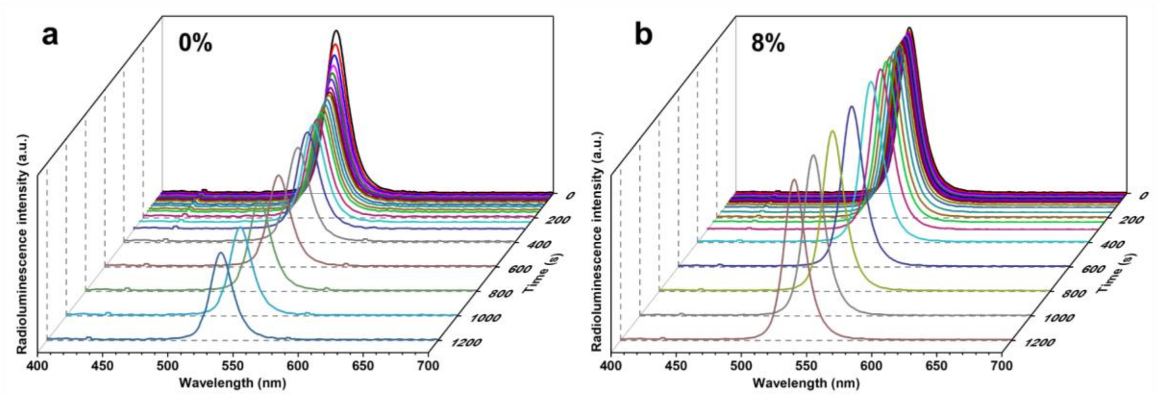

Figure S11. Radioluminescence spectra of undoped $CsPbBr_3$ NCs (a) and $CsPbBr_3$ NCs with 8% Ce^{3+} doped (b) under continuous X-ray irradiation.

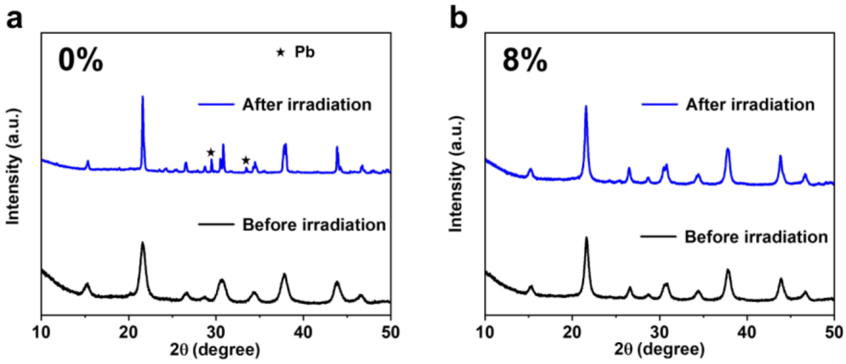

Figure S12. XRD patterns of (a) undoped and (b) Ce^{3+}-doped (8%) $CsPbBr_3$ NCs films before and after 3h X-ray irradiation (160 kV, 25 mA, MultiRad 160).

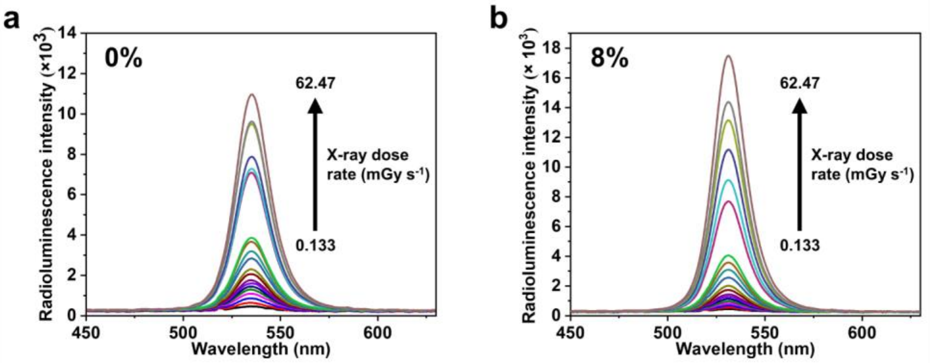

Figure S13. Radioluminescence spectra of undoped scintillator film (a) and scintillator film with 8% Ce^{3+} doping concentration (b) under various X-ray dose rates.

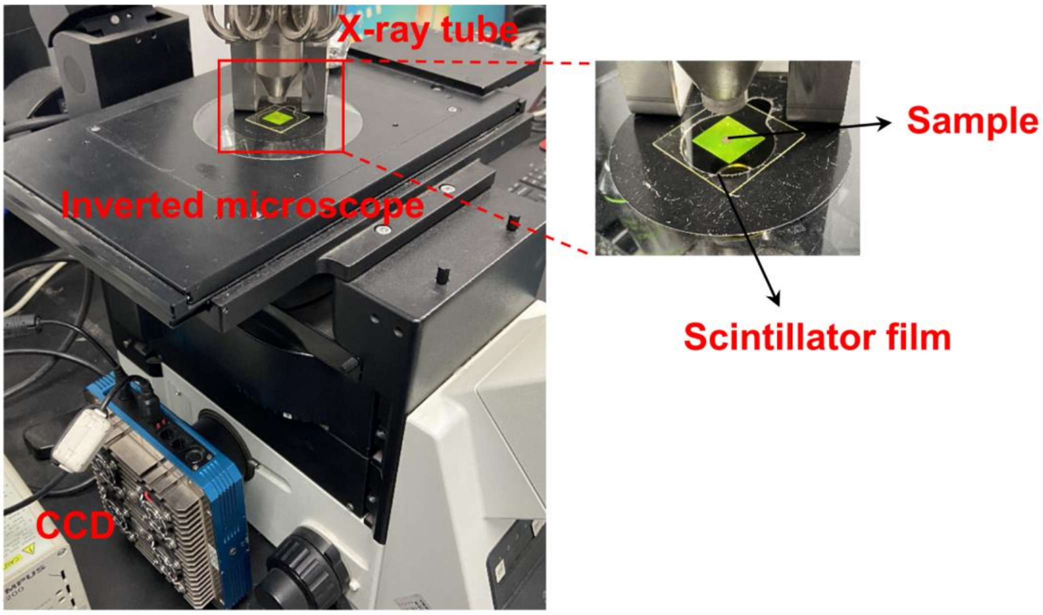

Figure S14. X-ray microscopic imaging system.

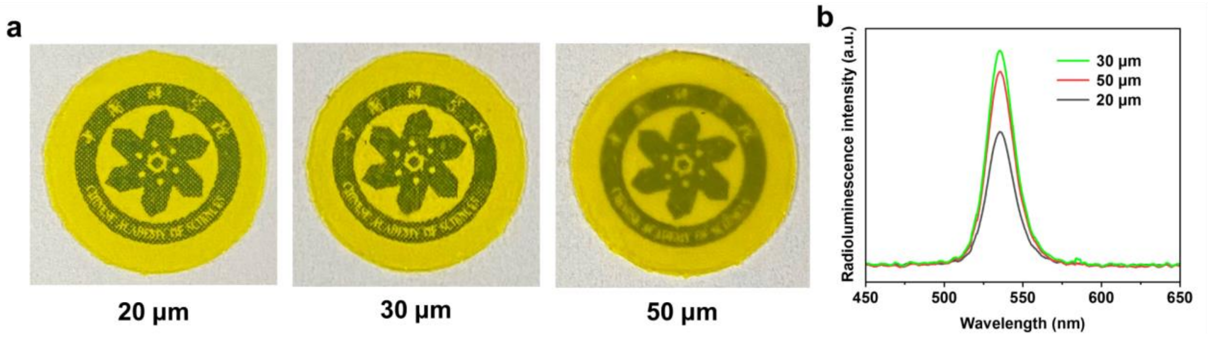

Figure S15. a) Photographs of scintillator films with the thickness of 20 μm, 30 μm and 50 μm on the top of the logo. b) Radioluminescence spectra of scintillator films with the thickness of 20 μm, 30 μm and 50 μm. Scintillator films with 20 μm and 30 μm thickness show similar transparency, while film with 50 μm thickness shows poor transparency. The scintillator film with 20 μm thickness suffer from its low X-ray absorption ability and 50 μm scintillator film limited by its strong self-absorption and low transparency. The scintillator film with 30 μm exhibit higher RL intensity and better transparency. So, we choose the optimal thickness of scintillator film is around 30 μm.

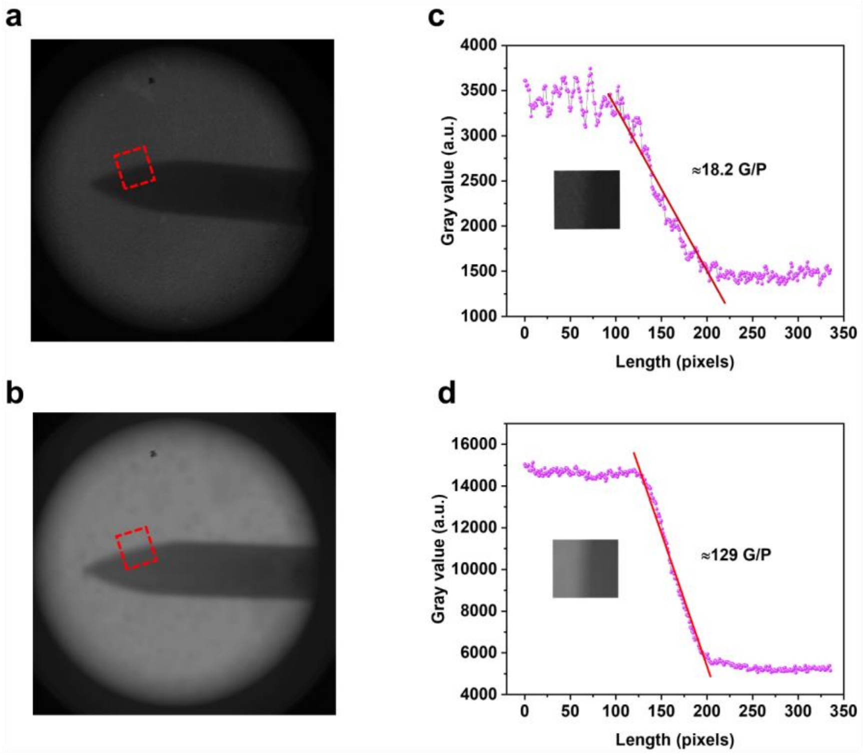

Figure S16. X-ray images of the needle and their edge diffusion function (ESF). X-ray images of the needle using the scintillator film prepared by (a) spin-coated and (b) suction filtration methods. c, d) ESF along the lines in the X-ray images (as shown in the inset).

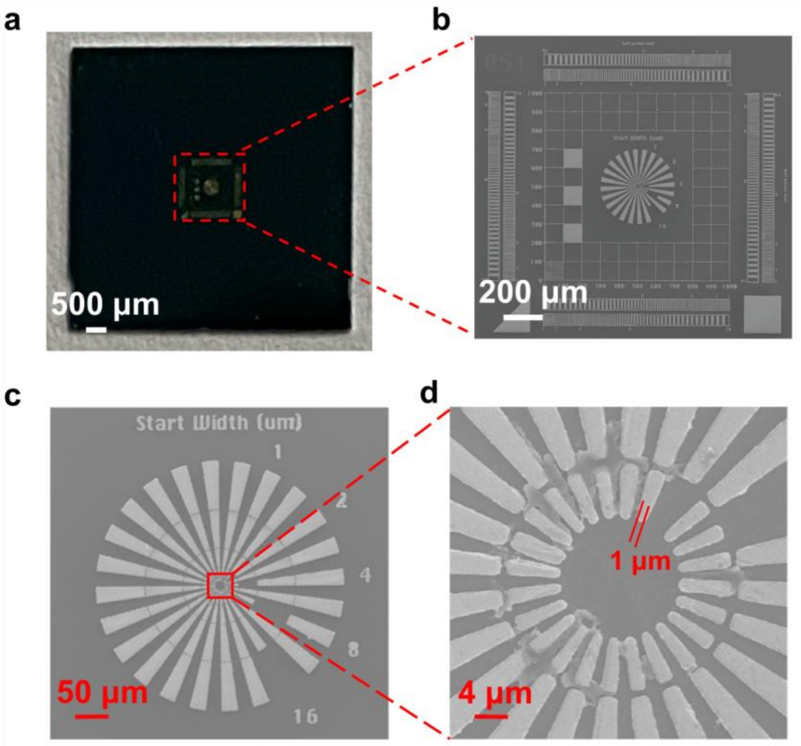

Figure S17. Characterization of the line pair card. a) Photograph and (b) overall SEM image of the line pair card. c) SEM images of the center circular area of the line pair card and (d) its enlarged view.
